# Effects of Social Vulnerability and Spatial Accessibility on COVID-19 Vaccination Coverage: A Census-Tract Level Study in Milwaukee County, USA

**DOI:** 10.3390/ijerph191912304

**Published:** 2022-09-28

**Authors:** Zengwang Xu, Bin Jiang

**Affiliations:** 1 Department of Geography, University of Wisconsin-Milwaukee, Milwaukee, WI 53201, USA; 2 Faculty of Engineering and Sustainable Development, Division of GIScience, University of Gävle, 801 76 Gävle, Sweden

**Keywords:** COVID-19, vaccination, social vulnerability, disparity, Milwaukee

## Abstract

COVID-19 vaccination coverage was studied by race/ethnicity, up-to-date doses, and by how it was affected by social vulnerability and spatial accessibility at the census-tract level in Milwaukee County, WI, USA. Social vulnerability was quantified at the census-tract level by an aggregate index and its sub-components calculated using the principal components analysis method. The spatial accessibility was assessed by clinic-to-population ratio and travel impedance. Ordinary least squares (OLS) and spatial regression models were employed to examine how social vulnerability and spatial accessibility relate to the vaccination rates of different doses. We found great disparities in vaccination rates by race and between areas of low and high social vulnerability. Comparing to non-Hispanic Blacks, the vaccination rate of non-Hispanic Whites in the county is 23% higher (60% vs. 37%) in overall rate (one or more doses), and 20% higher (29% vs. 9%) in booster rate (three or more doses). We also found that the overall social-vulnerability index does not show a statistically significant relationship with the overall vaccination rate when it is defined as the rate of people who have received one or more doses of vaccines. However, after the vaccination rate is stratified by up-to-date doses, social vulnerability has positive effects on one-dose and two-dose rates, but negative effects on booster rate, and the effects of social vulnerability become increasingly stronger and turn to negative for multi-dose vaccination rates, indicating the increasing challenges of high social vulnerability areas to multi-dose vaccination. The large negative effects of socio-economic status on the booster rate suggests the importance of improving general socio-economic conditions to promote multi-dose vaccination rates.

## 1. Introduction

Immunization is fundamental to prevent potential outbreaks of emerging or re-emerging infectious diseases or viruses [1,2,3,4]. A sufficient proportion of a population has to be vaccinated to achieve the herd immunity that is necessary to prevent population-level outbreaks. Structural barriers and individual-level beliefs and perceptions can interfere with vaccine uptake [5]. Vaccine hesitancy is influenced by many factors, which the World Health Organization conceptualized in a “3Cs” framework (i.e., complacency, confidence, and convenience), in which social vulnerability and spatial accessibility are two major factors [5,6]. Since the beginning of the COVID-19 vaccination campaign, a variety of efforts have been made to understand individual-level hesitancy and incentives in order to increase vaccination coverage [7,8,9,10,11,12]. Understanding disparities and inequities in vaccination coverage in different populations and neighborhoods is also crucial for effective vaccination strategies. This study focuses on the disparities in COVID-19 vaccination coverage by race/ethnicity and up-to-date doses and how social vulnerability and spatial accessibility affect the vaccination coverage in census tracts in Milwaukee County—a large urban county in the mid-western United States (U.S.).

Studies on the early stage of the COVID-19 vaccination campaign in the U.S. have found marked disparities and inequities in the vaccination coverage, and the populations of Blacks, Hispanics, and populations in rural areas and areas of high social vulnerability have been found to have lower and inequitable vaccination coverage [13,14,15,16,17,18,19]. Although with timely updates, the early studies on COVID-19 vaccination have many caveats. One of the major limitations in the national-level studies on racial and ethnic disparities is the lack of the consistent reporting and definition of race and ethnicity across the state health departments [17]. The studies have been affected by the prioritization of the COVID-19 vaccination campaign. In the beginning of the campaign prior to April 2021, populations of particular age groups, occupational exposures, and those with underlying health conditions were prioritized, and the prioritization was different in different jurisdictions [20,21]. Early studies have focused on the state or county levels and have not been able to address the neighborhood level, which would be more informative in planning effective vaccination strategies for local jurisdictions, as vulnerability and vaccination coverage vary within counties, especially those counties with large populations. The early studies have focused on the overall vaccination rate, which was defined as the rate of people who have received at least one dose of the COVID-19 vaccines. As the efficacy of the COVID-19 vaccines declines over time, two doses of vaccines are needed to be completely vaccinated, and a third and even fourth dose (i.e., booster) is recommended. Vaccination rates of different doses provide more details about vaccine hesitancy and vaccination coverage. With the COVID-19 vaccination records of residents in Milwaukee County as of 31 December 2021 from the Wisconsin Department of Health Statistics, this study investigates the disparities in the vaccination coverage by race/ethnicity and doses, and how social vulnerability and spatial accessibility affect the vaccination coverage at the neighborhood level down to the census tracts in Milwaukee County.

Social vulnerability refers to the many vulnerable social conditions that pose potential adverse effects to people’s exposure and recovery from hazards [22,23]. It has been considered a structural barrier to the coverage and equity of vaccination [13,14]. Spatial accessibility to vaccination facilities is another important structural barrier which interferes with the transfer of potential vaccination capability to the actual vaccine uptake [5,24]. The availability of vaccination facilities and the accessibility of the facilities are two components of spatial accessibility [24]. It has been found that increasing the availability of accessible and convenient vaccination locations can increase vaccination coverage and improve equity [25,26]. The COVID-19 vaccination campaign, under the federal partnership programs with pharmacies, has engaged many retail pharmacies or clinics to provide vaccinations, which should have increased the spatial accessibility of COVID-19 vaccination.

**Figure 1:**
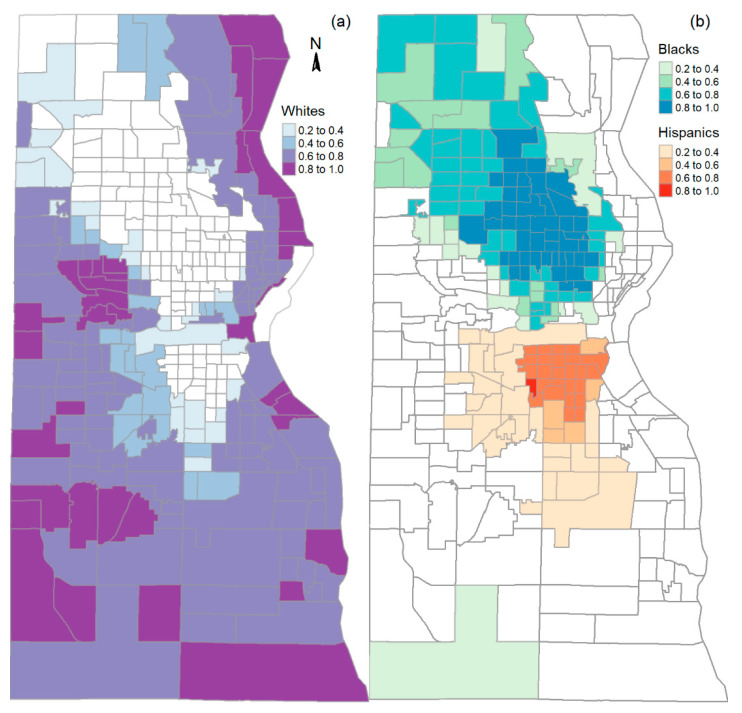
Spatial distribution of the proportions of non-Hispanic Whites (**a**), Blacks and Hispanics (**b**) in census tracts in Milwaukee County (based on 2020 census redistricting summary file).

Milwaukee County is the most populous county in the State of Wisconsin in the U.S. It has 939,490 residents according to the 2020 decennial census. Non-Hispanic Whites, Blacks, and other races, and Hispanics account, respectively, for 49%, 28%, 8%, and 16% of the total population. Seventy-four percent of the non-Hispanic other races are Asians. Milwaukee has been ranked one of the America’s most racially segregated metropolitan areas since 1970s, and all the key well-being measures of its Black community are consistently at or near the lowest level among the large metropolitan areas in the U.S. [27]. Figure 1 shows the spatial distribution of non-Hispanic Whites, Blacks, and Hispanics in Milwaukee County by mapping the proportion of the populations at census-tract level. We hypothesize that not only the spatial residential segregation patterns but also the social structural forces that have been at work play a role in COVID-19 vaccination coverage. Social vulnerability is employed to characterize the underlying social structural barriers to COVID-19 vaccination.

## 2. Materials and Methods

### 2.1. Index of Social Vulnerability

Many social economic and demographic variables have been included in computing social vulnerability indexes, though there is no consensus on a specific set of variables to be used as the standard for social vulnerability index (SVI). Twenty-five census-tract level variables were selected from the 2015–2019 5-year American Community Survey (ACS) and the 2020 decennial census redistricting summary file for Milwaukee County. The variables include all the 15 variables used to develop the social vulnerability index by the Centers of Disease Control and Prevention [23], supplemented by 10 other variables that may be relevant to vulnerability to COVID-19 infection and vaccination (Table 1). 

Principal components analysis (PCA) was the main method to construct social vulnerability index [22]. The PCA method was applied to the 25 variables, and five principal components (PCs) that have eigen values larger than one were extracted. All the 25 variables were standardized as z-scores for the PCA method. The z-score value of the median household income was inversed so that low value in median household income corresponds to high social vulnerability—the same interpretation as the value of other variables. The five PCs represent, collectively, 76% of the total variance in the 25 original variables (Table 1). The scores of the five PCs were summed to form the overall social vulnerability index of census tracts in Milwaukee County.

**Figure 2:**
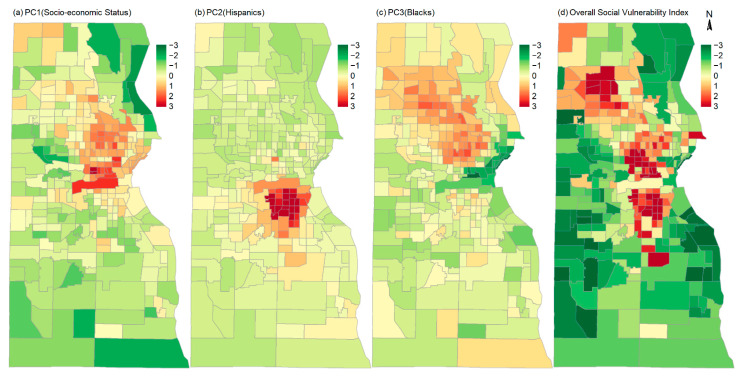
Spatial distribution of the score of the three major principal components (PCs): (**a**) PC1 representing general socio-economic status (SES); (**b**) PC2 representing Hispanics; (**c**) PC3 representing non-Hispanic Blacks; and (**d**) the value of the overall social vulnerability index (SVI) in census tracts in Milwaukee.

Each PC can be characterized by the variables to which it has high loading value. The variables and their loading values to each PC are listed in Table 1. Based on the high loading values (bolded in Table 1), PC1 is characterized as the vulnerability due to general socio-economic status (i.e., SES); PC2 is characterized as the vulnerability related to Hispanic population; and PC3 is characterized as the vulnerability related to non-Hispanic Blacks. These three PCs together account for 59% of the variance in the 25 variables (Table 1), and they all exhibit visually prominent spatial patterns (Figure 2). PC4 and PC5 do not have discernable spatial patterns and are not significant in most of the statistical models described in the later section. They were not included in the final statistical models. However, they were included in calculating the overall social vulnerability index (SVI).

**Figure 3:**
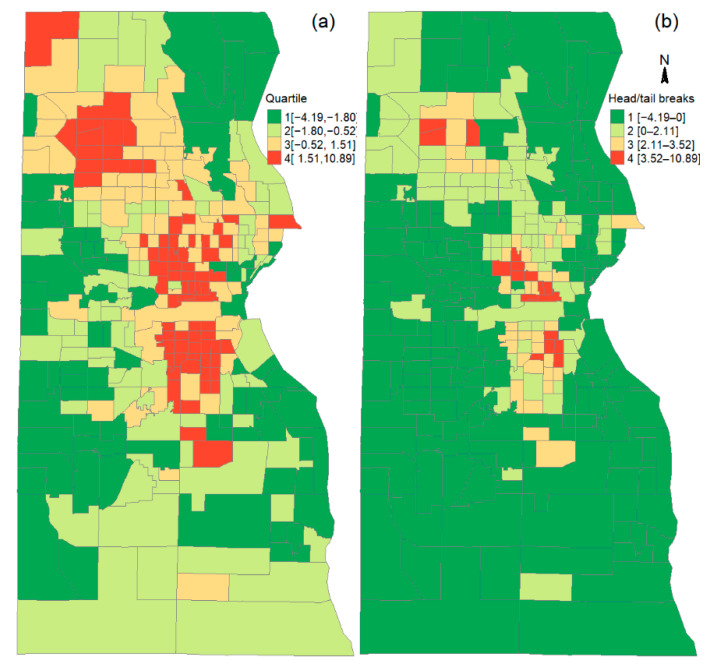
Spatial distribution of high and low social vulnerability areas based on (**a**) quartile and (**b**) head/tail breaks classification of the social vulnerability index. The red color (i.e., the fourth quarter or the large-value head class) represents high social vulnerability, and the green color (i.e., the first quarter or the small-value tail) represents low social vulnerability.

The overall SVI value of tracts was used to classify the tracts in Milwaukee County into high- and low-SVI areas. Two classification methods were employed: quartile and head/tail breaks classification [28,29]. The quartile method is a commonly used method. The head/tail breaks method is useful to classify data with right-skewed long tail distribution [28,30]. The head/tail breaks method can classify the social vulnerability index into as many as seven classes. The classes 4–7 have very small size and they are combined into one larger size class that has 17 census tracts. These 17 tracts have large SVI value and constitute class 4 in the head/tail breaks method to represent the high SVI areas. The spatial distribution of all these classes and their intervals resulting from both methods are shown in Figure 3. The tracts with social vulnerability index in the first quarter or the low-value tail class in head/tail breaks classification (i.e., the 1st class on Figure 3) constitute what we refer to as low vulnerability areas, and those tracts in the fourth quarter or the large-value head class in head/tail breaks classification (i.e., the 4th class on Figure 3) constitute the areas of high social vulnerability. The vaccination rates by race/ethnicity and doses were calculated in both the low and high social vulnerability areas, as well as in the whole county. 

### 2.2. Vaccination Rates by Race/Ethnicity, Doses, and in Areas of Different Social Vulnerability

**Figure 4:**
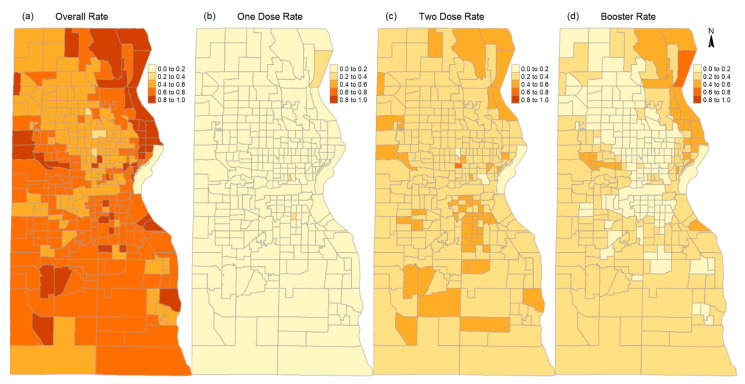
Age-adjusted vaccination rates in census tracts in Milwaukee: (**a**) overall rate; (**b**) one-dose rate; (**c**) two-dose rate; and (**d**) booster rate.

The vaccination rate of a population is defined as the rate of people who have been vaccinated. In many COVID-19 vaccination studies, people are considered vaccinated when they have received at least one dose of any of the Food and Drug Administration (FDA) authorized COVID-19 vaccine [21]. The vaccination rates in this study were stratified by the number of doses of the COVID-19 vaccines each person has received. Four vaccination rates were calculated, i.e., the overall vaccination rates of at least one dose ($r_{\geq 1}$), one dose only ($r_{1}$), two doses only ($r_{2}$), and three and more doses (or booster rate) of the vaccines ($r_{\geq 3}$); thus, $r_{\geq 1}=r_{1}+r_{2}+r_{\geq 3}$. These rates were calculated by race/ethnicity and in areas of high and local social vulnerability as well as in the whole county. Since the COVID-19 vaccines were initially authorized for people aged 18 years and older, the total population aged 18 years and older has been commonly used to calculate the vaccination rates [13]. The vaccination data in this study include people 5 years and older. Thus, the population of 5 years and older was used to calculate the vaccination rates in this study. At census-tract level, the vaccinated population was age-adjusted using the direct method [31]. The direct method uses the mean census-tract level population of different age groups in Milwaukee (based on the ACS 2015–2019 5-year estimates) as the standard population to project the expected number of vaccinated in census tracts where the census tracts had the same age structure as the standard population. The age adjustment was applied to the vaccinated populations of different doses. Figure 4 shows the age-adjusted vaccination rates in census tracts in Milwaukee. The spatial distribution patterns of the overall rate, two-dose rate, and booster rate show negative correlations with social vulnerability index (Figure 2), i.e., in general, high rate areas are those areas with low social vulnerability and low rate areas are those areas with high social vulnerability.

### 2.3. Spatial Accessibility to Vaccination Clinics

**Figure 5:**
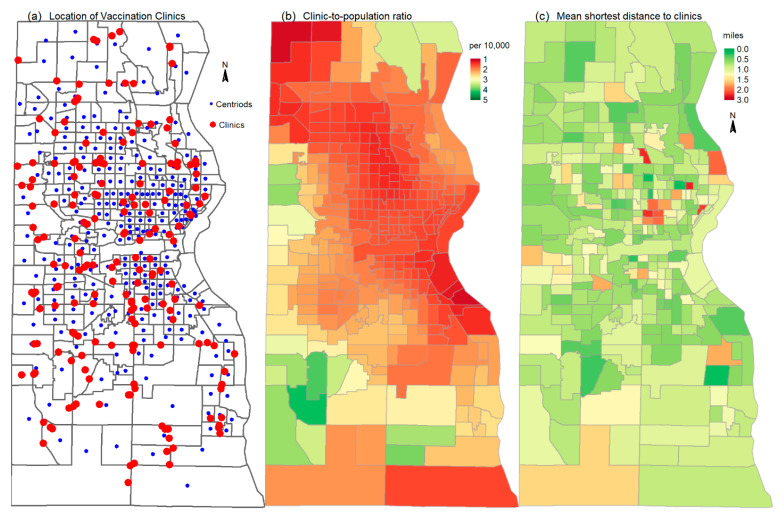
(**a**) Spatial location of the vaccination clinics, (**b**) tract-level clinic-to-population ratio, and (**c**) tract-level mean shortest travel distance to three nearest clinics.

According to the website vaccines.gov, there are 347 vaccination clinics within the Milwaukee Metropolitan area, and 187 within Milwaukee County. Most of them are retail pharmacies such as CVS, Walgreens, Walmart, and Pick-N-Save Pharmacies, and the like. All these retail pharmacies have emerged to provide preventative and acute care service in the United States since early 2000s [32]. The spatial accessibility to the vaccination clinics was assessed in terms of both availability (i.e., clinic-to-population ratio) and accessibility (i.e., travel impedance) using well-established methods [24,33]. The clinic-to-population ratio of a census tract was calculated as the mean ratio of the spatial density between the vaccination clinics and population [24]. Both the spatial densities of clinics and population were calculated using ArcGIS kernel density analysis tool using a 500-feet cell and a 3-mile searching radius. The spatial density of population was based on the centroids and population of the 2020 census blocks. The spatial density of clinics was based on the location of the vaccination clinics. The results of both densities are ArcGIS raster data. The clinic-density raster was divided by population-density raster to derive a clinic-to-population ratio raster, on which ArcGIS zonal statistics tool was applied to calculate the mean ratio of the raster cells in each census tract as the mean clinic-to-population ratio of the census tract. The travel impedance was calculated as the mean shortest travel distance from the population-weighted centroids of tracts to three nearest clinics along the roadway network in the whole Milwaukee Metro area, so that tracts along the Milwaukee County boundaries can access clinics outside the county. The population-weighted centroids of the census tracts were calculated using the population and centroids of census blocks from the 2020 census [34]. Figure 4 shows the location of the vaccination clinics and the population-weighted centroids of census tracts in Milwaukee County, and the tract level clinic-to-population ratio and the mean shortest distance to three nearest clinics. The tract level clinic-to-population ratio ranges 1.17–4.14 per 10,000 people. The clinic-to-population ratio map shows that majority of tracts have the ratio of 1–2 in 10,000, implying that, on average, 1–2 clinics are available for every 10,000 people in those areas. The tract-level mean shortest distance to clinics range from 0.25 to 2.59 miles. The distance of majority tracts is less than 1 mile. Only a few tracts have the distances greater than 1 mile, and these include the six contiguous tracts in the high vulnerability areas (Figure 5).

### 2.4. Modeling Vaccination Rates by Social Vulnerability and Spatial Accessibility

To examine the effects of social vulnerability and spatial accessibility on the vaccination coverage, the overall social vulnerability index and its three major components, as well as the two spatial accessibility measures, were included as the independent variables in ordinary least squares (OLS) regression and spatial regression models. The dependent variables are the natural logarithm of the age-adjusted vaccination rates for at least one dose ($r_{\geq 1}$), one dose only ($r_{1}$), two doses only ($r_{2}$), and three and more doses (or booster rate) ($r_{\geq 3}$) of the vaccines. OLS regression models were used firstly, and significant spatial autocorrelation was found in the residuals of the models. To account for the spatial autocorrelation in statistical models, two basic spatial regression models were considered: the spatial-lag model and spatial-error model [35,36]. Spatial-lag models account for the spatial autocorrelation in the dependent variables by a spatial autoregressive term of the dependent variable, while spatial-error models account for the spatial autocorrelation by a spatial autoregressive term of the residuals [36]. The spatial autoregressive terms specify how observations (or residuals) correlate in neighboring census tracts. Here, the spatial autoregressive terms are based on a queen-style contiguity, i.e., census tracts that share boundaries are considered neighbors, which is the minimum level of contiguity in specifying spatial autocorrelation in local neighborhoods [37]. Many other specifications of the spatial regression models are possible based on different statistical and theoretical assumptions [36]. We did not employ more complicated spatial regression models, as our interest was to correct the OLS regression models by accounting for spatial autocorrelation, rather than the particular spatial and statistical processes modeled by the other model specifications. The Lagrange multiplier diagnostics for spatial dependence in linear models showed that spatial-lag models are the better choice for most of the models. 

## 3. Results

### 3.1. Disparate Vaccination Rates by Race/Ethnicity, Doses, and Social Vulnerability

There exist significant disparities in vaccination rates by race/ethnicity, doses, and in areas of different level of social vulnerability in Milwaukee County (Table 2). The non-Hispanic other race (mainly Asians) has the highest overall vaccination rate ($r_{\geq 1}$) (77%). However, non-Hispanic other race has a small total population and they are scattered throughout Milwaukee without concentrated spatial residential patterns. The $r_{\geq 1}$ of non-Hispanic Whites is 60%, which is 23% higher than that of Blacks (37%), and 11% higher than that of Hispanics (49%). The disparities in the county-level overall vaccination rates ($r_{\geq 1}$) among the racial/ethnic groups were mainly due to the disparities in the booster rates ($r_{\geq 3}$). Non-Hispanic Whites has the highest booster rate (29%), which is 20% higher than that of Blacks (9%).

Another study has found a negative correlation between social vulnerability and COVID-19 vaccination rate, i.e., high social vulnerability areas have low vaccination rates [14]. Milwaukee has the similar negative correlation between social vulnerability and vaccination rates, especially when $r_{\geq 1}$ and $r_{\geq 3}$ are considered, and this relationship remains even when low and high social vulnerability areas were defined by different classification methods, i.e., quartile and head/tail breaks methods. The vaccination rates in the following analysis are based on the low and high social vulnerability areas defined by the quartile method, but similar patterns can be found in the vaccination rates of the high and low social vulnerability areas defined by the head/tail breaks classification method. In Table 2, the vaccination rates of the high and low social vulnerability areas defined by the head/tail breaks method are enclosed by round brackets. The high social vulnerability areas in Milwaukee County have an overall vaccination rate ($r_{\geq 1}$) which is 15% lower than that of low social vulnerability areas (45% vs. 60%) (Table 2). This large disparity is due to the large disparity in the booster rate ($r_{\geq 3}$) between the high and low social vulnerability areas (i.e., 10% vs. 28%). The low overall vaccination rates ($r_{\geq 1}$) in high social vulnerability areas (or high overall vaccination rates in low social vulnerability areas) is consistent for all racial/ethnicity groups except Hispanics, which has a slightly higher $r_{\geq 1}$ in high social vulnerability areas. For $r_{1}$ and $r_{2}$, high social vulnerability areas have slightly higher vaccination rates, except that the $r_{2}$ of Blacks has a slightly higher rate in low social vulnerability areas, and the $r_{2}$ of Whites has the same rate in high and low social vulnerability areas. The negative correlation between social vulnerability and the booster rate holds for every racial/ethnic group. 

### 3.2. Effects of Social Vulnerability and Spatial Accessibility on Vaccination Rates

The results of the models of vaccination rates by social vulnerability and spatial accessibility are presented in Table 3. It appears that the overall social vulnerability index is not significantly related to the overall vaccination rate (i.e., $r_{\geq 1}$). However, the overall social vulnerability index has significant but different effects on the vaccination rates of different doses, i.e., significant and positive effects on one-dose rate ($r_{1}$) and two-dose rate ($r_{2}$), and significant and negative effects on the booster rate ($r_{\geq 3}$). This implies that neighborhoods with a high overall social vulnerability index tend to have relatively high one and two-dose vaccination rates, but low booster rates. The effects of the overall social vulnerability index decline with the vaccination rates of increasing numbers of doses, which implies that multi-dose vaccination presents an increasing challenge for high social vulnerability areas. The clinic-to-population ratio has positive effects on the vaccination rates except $r_{1}$, which means that increasing clinics will improve the overall rate (i.e., $r_{\geq 1}$) across Milwaukee, the two-dose rate especially in high social vulnerability areas, and the booster rate especially in low social vulnerability areas, but increasing clinics has no significant effects on the one-dose rate.

The models between the vaccination rates and the three major components of social vulnerability provide more details. PC1 (i.e., SES) has significant negative effects on $r_{\geq 1}$, which is the result of the significant positive effects on the one-dose rate ($r_{1}$) and significant negative effects on the two-dose rate ($r_{2}$) and the booster rate ($r_{\geq 3}$). PC2 (i.e., Hispanics) has positive effects on $r_{\geq 1}$ (only significant in OLS), which is constituted by the significant positive effects on one-dose and two-dose rates but significant negative effects on booster rate. PC3 (i.e., Blacks) has similar effects as the PC1, except its effect on the $r_{2}$ is not significant. All three components have significant positive effects on one-dose rate but significant and stronger negative effects on booster rate. It implies that high social vulnerability neighborhoods (i.e., high vulnerability in general socio-economic status, and high percentage of Hispanics and Blacks) have lower coverage in booster rate, but relatively higher coverage in one-dose rate across Milwaukee county. The effects of all the three components decline with the vaccination rates of increasing number of doses, confirming the greater challenge of multi-dose vaccination for people in high social vulnerability areas. Among the three components, the general socio-economic status has the largest negative effects on the booster rate, implying the importance of improving general socio-economic conditions to promote the multi-dose vaccination. The clinic-to-population ratio is only significant in modeling two-dose rate with the Hispanics component. This implies that increasing the availability of vaccination clinics will improve two-dose rate, especially in areas with high percentage of Hispanics. 

The Hispanics component has significant positive and strongest effects (comparing to SES and Blacks) on one and two-dose rates ($r_{1}\;and\;r_{2}$) and negative and lowest effects (again comparing to SES and Blacks) on booster rate ($r_{\geq 3}$). This could be a result of the late start of vaccination, and Hispanics with two doses of vaccines have yet to receive the booster during the study period. It also could be that receiving three or more doses of vaccines in the required time intervals has presented an even greater challenge or hesitancy for Hispanics. However, Hispanics are a rapidly growing minority in Milwaukee and have demonstrated a unique relationship to COVID-19 vaccination coverage. 

## 4. Discussion

The shortest travel distance was never a significant factor in any models. This is consistent with the observation that the travel impedance often loses validity in congested urban areas in spite of it being the most popular measure of spatial accessibility to care [24]. This might be because the density of the vaccination facilities is high enough in urban areas so the variance in travel distance to the facilities is no longer large enough to be a factor to explain the variance in vaccination rates. The longest mean shortest distance from the centroid of a census tract to its three nearest clinics is only 2.6 miles in Milwaukee. The two spatial accessibility factors do not have a significant correlation, which is consistent with the finding that high facility density is not correlated with low average driving distance at the county level [38]. A simple exploration of the six tracts with travel distances larger than 2 miles (all have relative low clinic-to-population ratios) finds that three have more than a 90% minority population (the sum of Hispanics, Blacks, and Asians), two have majority renters and multi-unit households, and one has very high median household income ($142,917). These tracts allow a glimpse into the interaction between social vulnerability and spatial accessibility in the neighborhoods in Milwaukee.

One limitation of this study is that the vaccination capacity or other aspatial accessibility factors of the vaccination clinics were not considered due to the data availability. The vaccination clinics in this study do not include the mobile vaccination clinics that were temporally set up in large employment centers by the health departments of the cities in Milwaukee County. This study was based on the most detailed vaccination data and most up-to-date social demographics data. However, a limitation is still the accuracy of the vaccination records from the state health department and their consistency with the social demographic data from the censuses. A large number of people in vaccination records (about 10%) do not have a geocode. Both the American Community Survey (ACS) and decennial census data have been used in this study. ACS only provides population by age and race for non-Hispanic Whites and Hispanics, while the 2020 decennial census redistricting program only provides the total population by race, and population by age for 18 years and older. The data from these two datasets are highly correlated but with small discrepancies, which do not affect our conclusions. Milwaukee is an urban county with almost one million residents, but it is a unique place in terms of its racial residential segregation patterns, which should be considered when this study is compared.

## 5. Conclusions

This study contributes an intra-city neighborhood-level analysis on the disparities in COVID-19 vaccination coverage and the effects of social vulnerability and spatial accessibility on vaccination coverage. The COVID-19 vaccination coverage in Milwaukee has exhibited great disparities by race/ethnicity, up-to-date doses, and in areas of different social vulnerability. The vaccination rate of non-Hispanic Whites was more than 20% larger than that of non-Hispanic Blacks. Comparing to low social vulnerability areas, high social vulnerability areas have significantly lower booster rates. At the intra-city neighborhood-level in Milwaukee, the overall social vulnerability index does not have statistically significant relationship with the overall vaccination rate ($r_{\geq 1}$) when it is defined as the rate of people who have received one or more doses of vaccines. However, when the vaccination rates are stratified by different up-to-date doses, the vaccination rates of different doses reveal more complex relations between the vaccination rates and social vulnerability and spatial accessibility at neighborhood level. Social vulnerability has positive effects on one-dose and two-dose rates, but negative on booster rates, and high social vulnerability areas exhibited increasing challenges/hesitancy to multi-dose vaccination. Increasing spatial accessibility to vaccination facilities will help Hispanic neighborhoods improve the two-dose rate, but spatial accessibility is no longer a significant factor for booster rate. Our results also show the importance of improving general socio-economic conditions in promoting multi-dose vaccination coverage. As the efficacy of many vaccines will decline over time and the multi-dose vaccination becomes commonplace, this study confirms the hesitancy of multi-dose vaccine in high social vulnerability areas at the intracity level, as well as the importance to study vaccination rates of different up-to-date doses.

## Figures and Tables

**Table 1:** The results of the PCA analysis on the census-tract level variables selected from 2015–2019 5-year American Community Survey and 2020 decennial census redistricting summary file (the bold loading values are the high loading of the variables used to characterize each principal component).

Eigen Value & Variance of the Principal Components					
	Principal Components				
	1	2	3	4	5
Eigen Value	6.48	4.47	3.79	2.26	2.04
Proportion Var	0.26	0.18	0.15	0.09	0.08
Cumulative Var	0.26	0.44	0.59	0.68	0.76
**Variables and Their Loadings to the Principal Components**					
people whose income is below poverty in the past 12 months (%)	**0.80**	0.27	0.25	0.08	0.28
5+ years who speak English less than well (%)	0.02	**0.90**	0.04	0.24	−0.01
25+ years with less than high school education (%)	0.40	**0.77**	0.34	0.05	−0.04
civilian noninstitutionalized population with disability (%)	**0.54**	0.04	0.20	−0.08	−0.63
median household income in past 12 months in 2019 ($)	**0.80**	0.30	0.21	0.01	−0.04
unemployed civilian labor force (%)	0.54	0.05	**0.62**	−0.03	−0.03
renter occupied units (%)	**0.88**	0.20	−0.10	0.01	0.15
multi-unit housing units (%)	**0.73**	0.23	−0.46	0.04	0.11
mobile homes (%)	−0.02	0.04	0.01	0.16	−0.24
16+ years workers without vehicle (%)	**0.80**	0.03	0.14	−0.06	0.20
16+ years workers who take public transit (%)	**0.74**	−0.04	0.30	−0.07	0.10
single parent households (%)	0.42	0.20	**0.82**	−0.02	0.05
3+ years enrolled in schools (%)	0.30	0.10	0.20	0.17	**0.78**
occupied housing units with more than one person per room (%)	0.21	**0.59**	0.37	0.35	0.14
population with no health insurance coverage (%)	0.25	**0.87**	0.10	−0.12	0.06
households with no internet access (%)	**0.68**	0.45	0.31	−0.04	−0.18
people in female householder families with no health insurance (%)	0.24	**0.49**	0.47	−0.21	−0.02
population 65 years and older (%)	−0.33	−0.34	−0.29	−0.01	−0.62
population 17 years and younger (%)	0.03	0.32	**0.87**	0.06	0.02
Hispanic population * (%)	−0.07	**0.95**	−0.09	−0.09	0.01
non-Hispanic black population * (%)	0.58	−0.26	**0.69**	−0.05	0.04
Asian population * (%)	−0.02	0.01	0.02	**0.98**	0.02
population of non-Hispanic other race * (%)	−0.08	0.00	−0.09	**0.97**	0.02
group quarter population * (%)	0.23	−0.14	−0.43	0.06	**0.59**
vacant housing units * (%)	**0.75**	−0.02	0.36	−0.09	0.11

* based on 2020 decennial census redistricting summary file.

**Table 2:** Vaccination rates (%) by race/ethnicity and number of doses in the whole Milwaukee County and in areas of high and low social vulnerability.

		Vaccination Rates (%)											
Number of Doses		One or More Doses			One Dose			Two Doses			Three or More Doses		
Areas		Whole County	High SVI Areas	Low SVI Areas	Whole County	High SVI Areas	Low SVI Areas	Whole County	High SVI Areas	Low SVI Areas	Whole County	High SVI Areas	Low SVI Areas
Race	Blacks	37	34 (29)	43 (41)	6	6 (6)	6 (6)	22	21 (18)	25 (24)	9	7 (5)	12 (11)
	Hispanics	49	51 (47)	47 (47)	7	8 (8)	6 (7)	32	34 (32)	28 (29)	10	8 (7)	13 (12)
	Other	77	65 (53)	82 (82)	8	9 (7)	7 (8)	44	45 (39)	40 (43)	25	11 (7)	36 (32)
	Whites	60	50 (37)	61 (61)	5	6 (4)	5 (5)	26	26 (22)	26 (26)	29	18 (10)	30 (30)
Total population		53	45 (40)	60 (58)	6	7 (6)	5 (5)	27	28 (26)	27 (27)	20	10 (7)	28 (25)

Note: The high social vulnerability index (SVI) areas are defined as the tracts with large SVI values that are either in the fourth quartile or the large-value head class in head/tail breaks classification, and the low SVI areas are defined as the tracts with small SVI values that are either in the first quartile or the small-value tail class in head/tail breaks classification. Figure 3 shows the spatial distribution of these areas. The vaccination rates of the high and low SVI areas that are based on head/tail breaks classification are enclosed with round brackets.

**Table 3:** Results of OLS and spatial-lag models of the vaccination rates by social vulnerability and spatial accessibility.

	Dependent Variables							
	$log\left(r_{\geq 1}\right)$		$log(r_{1})$		$log(r_{2})$		$log(r_{\geq 3})$	
	*OLS*	*Lag*	*OLS*	*Lag*	*OLS*	*Lag*	*OLS*	*Lag*
Overall social vulnerability index	−0.007	−0.001	0.059 ***	0.046 ***	0.025 ***	0.020 ***	−0.085 ***	−0.024 ***
Clinic-to-population ratio	0.134 ***	0.088 ***	0.038	0.041	0.142 ***	0.110 ***	0.189 ***	0.073 **
Shortest travel distance	−0.021	−0.024	−0.050	−0.050	−0.038	−0.035	0.015	−0.009
Adjusted/pseudo R^2^	0.093	0.254	0.126	0.178	0.096	0.175	0.295	0.623
ρ		0.489 ***		0.291 ***		0.344 ***		0.745 ***
PC1 (SES)	−0.106 ***	−0.089 ***	0.076 ***	0.061 ***	−0.038 ***	−0.035 ***	−0.287 ***	−0.213 ***
PC2 (Hispanics)	0.022 *	0.016	0.105 ***	0.081 ***	0.085 ***	0.078 ***	−0.117 ***	−0.080 ***
PC3 (Blacks)	−0.073 ***	−0.055 ***	0.067 ***	0.059 ***	−0.007	−0.006	−0.225 ***	−0.147 ***
Clinic-to-population ratio	0.020	0.016	0.052	0.052	0.076 ***	0.072 ***	−0.048	−0.053
Shortest travel distance	−0.004	−0.009	−0.034	−0.038	−0.017	−0.018	0.023	0.010
Adjusted/pseudo R^2^	0.295	0.328	0.145	0.187	0.212	0.228	0.667	0.714
ρ		0.228 ***		0.246 ***		0.082		0.384 ***

*Note:* ρ is the spatial lag component. * *p* < 0.1, ** *p* < 0.05, *** *p* < 0.01.

## Data Availability

Not applicable.
